# Granisetron compared with prednisolone plus metopimazine as anti-emetic prophylaxis during multiple cycles of moderately emetogenic chemotherapy

**DOI:** 10.1038/sj.bjc.6690372

**Published:** 1999-05-01

**Authors:** T Sigsgaard, J Herrstedt, L J Andersen, H Havsteen, S W Langer, A-G Kjærbøl, H Lund, M Kjær, P Dombernowsky

**Affiliations:** Department of Oncology, Herlev Hospital, University of Copenhagen, Denmark; Department of Oncology, Aalborg Hospital, Aalborg, Denmark; Department of Oncology, Vejle Hospital, Denmark; Department of Internal Medicine P, Bispebjerg Hospital, Denmark

**Keywords:** anti-emetics, dopamine D_2_ antagonist, granisetron, metopimazine, multiple cycles, prednisolone

## Abstract

This randomized, double-blind, double-dummy parallel study compared the anti-emetic efficacy and tolerability of the serotonin antagonist granisetron with prednisolone plus the dopamine D_2_ antagonist metopimazine during nine cycles of moderately emetogenic chemotherapy. Chemotherapy naive women with stage I or II breast cancer scheduled to intravenous cyclophosphamide, fluorouracil and methotrexate or cyclophosphamide, epirubicin and fluorouracil every 3 weeks were included. Patients received a single intravenous dose of granisetron 3 mg or a 3-day oral treatment with prednisolone 25 mg once a day plus metopimazine 30 mg four times a day. A total of 223 women were enrolled and 218 patients (97.8%) were evaluable for efficacy. Granisetron (*n* = 109) was superior to prednisolone plus metopimazine (*n* = 109) in the prophylaxis of acute nausea and vomiting during the first cycle of chemotherapy (*P* < 0.001) and prednisolone plus metopimazine was superior on days 2–5 (*P* = 0.002). Overall, granisetron was superior on days 1–5 (*P* = 0.009). The median number of cycles completed with granisetron was five (95% confidence interval 4–6) compared with two (95% confidence interval 2–2) for prednisolone plus metopimazine (*P* = 0.0019). Constipation and rash were reported more frequently with granisetron (*P* < 0.001 and *P* = 0.043 respectively) and palpitations more frequently with prednisolone plus metopimazine (*P* = 0.015). In conclusion, the number of cycles completed with granisetron was significantly higher than the number completed with prednisolone plus metopimazine, but the anti-emetic efficacy of both treatments declined during multiple cycles of moderately emetogenic chemotherapy. © 1999 Cancer Research Campaign


*British Journal of Cancer* (1999) **80**(3/4), 412–418
© 1999 Cancer Research Campaign
Article no. bjoc.1998.0372

### Granisetron compared with prednisolone plus metopimazine as anti-emetic prophylaxis during multiple cycles of moderately emetogenic chemotherapy

**T Sigsgaard[1], J Herrstedt[1], LJ Andersen[2], H Havsteen[3], SW Langer[4], A-G Kjærbøl[1], H Lund[1], M Kjær[2] and P Dombernowsky[1]**

[1]Department of Oncology, Herlev Hospital, University of Copenhagen, Denmark; [2]Department of Oncology, Aalborg Hospital, Aalborg, Denmark; [3]Department of Oncology, Vejle Hospital, Denmark; [4]Department of Internal Medicine P, Bispebjerg Hospital, Denmark

**Summary** This randomized, double-blind, double-dummy parallel study compared the anti-emetic efficacy and tolerability of the serotonin antagonist granisetron with prednisolone plus the dopamine $D_2$ antagonist metopimazine during nine cycles of moderately emetogenic chemotherapy. Chemotherapy naive women with stage I or II breast cancer scheduled to intravenous cyclophosphamide, fluorouracil and methotrexate or cyclophosphamide, epirubicin and fluorouracil every 3 weeks were included. Patients received a single intravenous dose of granisetron 3 mg or a 3-day oral treatment with prednisolone 25 mg once a day plus metopimazine 30 mg four times a day. A total of 223 women were enrolled and 218 patients (97.8%) were evaluable for efficacy. Granisetron ($n$ = 109) was superior to prednisolone plus metopimazine ($n$ = 109) in the prophylaxis of acute nausea and vomiting during the first cycle of chemotherapy ($P$ < 0.001) and prednisolone plus metopimazine was superior on days 2–5 ($P$ = 0.002). Overall, granisetron was superior on days 1–5 ($P$ = 0.009). The median number of cycles completed with granisetron was five (95% confidence interval 4–6) compared with two (95% confidence interval 2–2) for prednisolone plus metopimazine ($P$ = 0.0019). Constipation and rash were reported more frequently with granisetron ($P$ < 0.001 and $P$ = 0.043 respectively) and palpitations more frequently with prednisolone plus metopimazine ($P$ = 0.015). In conclusion, the number of cycles completed with granisetron was significantly higher than the number completed with prednisolone plus metopimazine, but the anti-emetic efficacy of both treatments declined during multiple cycles of moderately emetogenic chemotherapy.

**Keywords:** anti-emetics; dopamine $D_2$ antagonist; granisetron; metopimazine; multiple cycles; prednisolone

The anti-emetic efficacy and tolerability of 5-hydroxytryptamine $(\text{serotonin})_3$ antagonists, dopamine $D_2$ receptor antagonists and steroids have been studied in patients receiving moderately emetogenic chemotherapy. Though patients receive multiple cycles of chemotherapy, the vast majority of these trials included only patients during the initial one or two cycles of chemotherapy.

Corticosteroids alone or combined with other anti-emetics are often used in the treatment of nausea and vomiting induced by moderately emetogenic chemotherapy. Most trials have used dexamethasone, whereas methylprednisolone and prednisolone have been used less frequently, but there is no evidence that differences in efficacy or toxicity exist between different corticosteroids (Cersosimo and Karp, 1986). The optimum dose and schedule of steroids has not yet been defined (Aapro, 1991). As single agents, corticosteroids are superior to placebo (Pollera et al, 1989) and equal or superior to metoclopramide during the first cycle of moderately emetogenic chemotherapy (Markman et al, 1984; Ibrahim et al, 1986; Roila et al, 1988). The combination of dexamethasone and a dopamine $D_2$ antagonist is also an effective and widely used anti-emetic regimen in moderately emetogenic chemotherapy. Metopimazine is a dopamine $D_2$ antagonist with anti-emetic activity superior to placebo (Moertel and Reitemeier, 1973; Israel and Rodary, 1978) and equal to prochlorperazine (Moertel and Reitemeier, 1973) when given in oral doses of 10–15 mg three times a day. Metopimazine is safe in oral doses of 30 mg four times a day (Herrstedt et al, 1997) and the anti-emetic effect seems to be increased in high doses (Israel and Rodary, 1978; Vallejo et al, 1988; Clavel et al, 1993). In contrast to other dopamine $D_2$ antagonists metopimazine has no extrapyramidal side-effects (Vallejo et al, 1988; Herrstedt et al, 1997).

The serotonin antagonist ondansetron is superior or equal to metoclopramide during the initial one or two cycles of moderately emetogenic chemotherapy (Bonneterre et al, 1990; Kassa et al, 1990; Marschner et al, 1991) but it is still debated if the anti-emetic efficacy of the serotonin antagonists exceeds that of the corticosteroids (Jones et al, 1991; Italian Group for Antiemetic Research, 1995*b*). The efficacy of the serotonin antagonists ondansetron, granisetron and tropisetron has been compared in several trials and no significant differences have been shown (Campora et al, 1994; Gebbia et al, 1994; Stewart et al, 1995).

The efficacy of anti-emetic treatment during multiple cycles of moderately emetogenic chemotherapy has only been evaluated in few trials and is not fully elucidated. Conclusions are not concordant as different methodology and different statistical analyses have been used. Some studies demonstrated a decrease in anti-emetic efficacy (Martin et al, 1992; Soukop et al, 1992) whereas others found sustained efficacy (Blijham, 1992; de Wet et al, 1993; Kaizer et al, 1994; Italian Group for Antiemetic Research, 1995*a*; Silva et al, 1996).

*Received 27 May 1998*
*Revised 20 October 1998*
*Accepted 5 November 1998*

*Correspondence to:* Tine Sigsgaard, Department of Internal Medicine F, Gentofte Hospital, Niels Andersens Vej 65, 2900 Hellerup, Denmark

**Table 1** Patient characteristics ($n$ = 223)

| | No. of patients (%) | |
|---|---|---|
| | **Granisetron** | **Prednisolone plus metopimazine** |
| No. of patients | 112 | 111 |
| Age (years) | | |
| Median | 47 | 46 |
| Range | 26–68 | 33–69 |
| Performance status | | |
| 0 | 109 | 108 |
| 1 | 3 | 3 |
| 2 | 0 | 0 |
| Chemotherapy | | |
| Cyclophosphamide, methotrexate and fluorouracil | 101 (90.2) | 102 (91.9) |
| Cyclophosphamide, epirubicin and fluorouracil | 11 (9.8) | 9 (8.1) |

Cyclophosphamide-containing chemotherapy during multiple cycles is widely used as adjuvant treatment in patients with breast cancer (Early Breast Cancer Trialists' Collaborative Group, 1992). The efficacy and tolerability of anti-emetic treatment during a standard regimen of nine courses of adjuvant cyclophosphamide-based chemotherapy has not previously been investigated. This randomized, double-blind study compares the anti-emetic activity and tolerability of granisetron with the combination of prednisolone plus metopimazine during nine courses of cyclophosphamide-based chemotherapy.

### PATIENTS AND METHODS

### Patients

Women with histologically confirmed stage I or II breast cancer scheduled for nine cycles of adjuvant chemotherapy were eligible. Other inclusion criteria were age between 18 and 70 years and a performance status of 0–2 according to WHO criteria (World Health Organization, 1979). Exclusion criteria were prior chemotherapy, a peptic ulcer or diabetes, gastrointestinal obstruction, nausea or vomiting within 24 h before the first cycle of chemotherapy and anti-emetic therapy (including steroids) during the week before entry. The use of benzodiazepines for night-time sedation was allowed.

### Chemotherapy

Chemotherapy consisted of cyclophosphamide 600 mg $m^{-2}$, methotrexate 40 mg $m^{-2}$ and fluorouracil 600 mg $m^{-2}$ (CMF), or cyclophosphamide 600 mg $m^{-2}$, epirubicin 60 mg $m^{-2}$ and fluorouracil 600 mg $m^{-2}$ (CEF) given intravenously every 3 weeks and planned for nine cycles. Patients treated with radiotherapy to the chest wall and axillary lymph nodes received cyclophosphamide 850 mg $m^{-2}$ during that period (usually cycles 2 and 3).

### Design of the study

A randomized, double-blind, placebo-controlled (double-dummy) parallel design was used. Patients were randomized in blocks of ten and stratified for centre and chemotherapy (CMF or CEF).

### Anti-emetic treatment

Patients were randomly assigned to a single intravenous dose of granisetron 3 mg (or placebo) diluted in 100 ml of saline given as a 5–15 min infusion starting 15 min before chemotherapy, or a 3-day oral treatment with prednisolone 25 mg once a day plus metopimazine 30 mg four times a day. The first dose of prednisolone and metopimazine (or identical-appearing placebo tablets) was given 30 min before the start of chemotherapy, the second dose of metopimazine (or placebo) 30 min before dinner, and the third just before bedtime. On days 2 and 3, metopimazine (or placebo) was taken 30 min before breakfast, lunch and dinner and just before the patient went to bed. Prednisolone (or placebo) was administered together with the first dose of metopimazine (or placebo). The study medication was given in a plastic container with a separate compartment for each dose. The container was returned at the next chemotherapy cycle and the number of unused study tablets counted. If, for any reason, the patients did not take all the tablets, they were asked to explain the reasons on their diary card.

### Assessment of anti-emetic efficacy

Following chemotherapy, patients recorded on days 1–5 on the diary cards the number of vomiting episodes and dry retches, and the severity of nausea and other adverse events. Any vomit productive of liquid or a dry retch was considered a single emetic episode. The severity of nausea and other adverse events were assessed on a graded scale as: 'none', 'mild', 'moderate', or 'severe'. Complete response (CR) was defined as no emetic episodes and no nausea or only mild nausea; major response (MR) as one emetic episode and/or moderate to severe nausea; minor response (mR) as two to four emetic episodes, and failure as five or more emetic episodes.

On the diary card day 5 after each cycle of chemotherapy, patients were instructed to tick off one of the following two possibilities: 'I have been satisfied with the anti-emetic treatment and want to continue with the same anti-emetic treatment during the next cycle of chemotherapy' or 'I have not been satisfied with the anti-emetic treatment and want another anti-emetic treatment during the next cycle'.

During cycles 1 and 2, and thereafter only when necessary, a research nurse called patients on days 2 and 5 to ensure that the study medication was taken and the diary cards completed. To assess the frequency of anticipatory nausea and vomiting, patients were again called 3 days before the next cycle of chemotherapy and reminded to record the number of vomiting episodes and dry retches, and grade nausea on days 1–3 before the next course. The diary card was returned at the following visit.

Patients were withdrawn from the study if they had had five or more emetic episodes on one or more days during the study period, or if they had ticked off on the diary card that they were not satisfied with the anti-emetic treatment. Patients receiving rescue medication were also withdrawn. If chemotherapy was changed, e.g. because of progressive disease, the patients were also withdrawn.

### Ethical considerations

The study was conducted according to the Helsinki II Declaration and was approved by the local Scientific Ethics Committees and by the Danish Medical Health Authorities. Written informed consent was mandatory.

*© Cancer Research Campaign 1999*

**Table 2** Off study reasons, cumulative protection rates and conditional protection rates among 218 patients receiving adjuvant chemotherapy and treated with granisetron or prednisolone plus metopimazine during cycles 1–9

| | Cycle number | | | | | | | | | |
|---|---|---|---|---|---|---|---|---|---|---|
| | 1 | 2 | 3 | 4 | 5 | 6 | 7 | 8 | 9 | All cycles |
| Total no. of patients | 218 | 155 | 118 | 98 | 83 | 68 | 57 | 52 | 49 | |
| Granisetron | | | | | | | | | | |
| No. of patients | 109 | 84 | 71 | 59 | 52 | 41 | 36 | 32 | 30 | |
| Failure | 17 | 4 | 4 | 4 | 5 | 3 | 1 | 0 | 2 | 40 |
| Not satisfied | 8 | 6 | 5 | 3 | 4 | 1 | 2 | 1 | 0 | 30 |
| Other reasons[a] | 0 | 3 | 3 | 0 | 2 | 1 | 1 | 1 | 0 | 11 |
| Cumulative protection rates | 0.77 | 0.68 | 0.59 | 0.52 | 0.43 | 0.39 | 0.36 | 0.35 | 0.32 | |
| Conditional protection rates | 0.77 | 0.88 | 0.87 | 0.88 | 0.83 | 0.90 | 0.92 | 0.97 | 0.93 | |
| Prednisolone plus metopimazine | | | | | | | | | | |
| No. of patients | 109 | 71 | 47 | 39 | 31 | 27 | 21 | 20 | 19 | |
| Failure | 34 | 22 | 5 | 7 | 4 | 4 | 1 | 1 | 1 | 79 |
| Not satisfied | 3 | 2 | 1 | 1 | 0 | 2 | 0 | 0 | 0 | 9 |
| Other reasons[a] | 1 | 0 | 2 | 0 | 0 | 0 | 0 | 0 | 0 | 3 |
| Cumulative protection rates | 0.66 | 0.44 | 0.38 | 0.30 | 0.26 | 0.21 | 0.20 | 0.19 | 0.18 | |
| Conditional protection rates | 0.66 | 0.66 | 0.87 | 0.79 | 0.87 | 0.78 | 0.95 | 0.95 | 0.95 | |

[a]These included cessation of chemotherapy (four patients), concomitant medication with benzodiazepines or corticosteroids (four patients), or other reasons (six patients).

### Statistical analysis

The number of patients to be enrolled in the trial was calculated on the assumption that CR or MR on day 1 during the first cycle of chemotherapy would be achieved in 60% of patients with prednisolone plus metopimazine, and in 80% of those treated with granisetron. Using a two-sided, 5% level test and a power of 0.8 it was estimated that 91 evaluable patients in each treatment arm was required. We therefore decided to include 220 patients.

The Mann–Whitney *U*-test was used to compare the number of patients obtaining CR, MR, mR and failure on day 1 (acute), days 2–5 (delayed) and days 1–5 (overall) in cycle 1. For days 2–5 and days 1–5 the analyses were based on the severity of nausea recorded on the worst day within the period and, for emesis both on the number of emetic episodes on the worst day in the period and on the total number of emetic episodes in the period.

Maintenance of emetic control during cycles 1–9 (not failure, satisfied with the anti-emetic treatment, and no rescue medication) was calculated by the Kaplan–Meier method (cumulative emetic control) and compared with the log-rank test. Patients going off study due to other reasons were regarded as censored. The number of completed cycles was compared with the log-rank test. The maintenance of emetic control was also calculated based on conditional probabilites (De Wit et al, 1996). The condition was that failure of anti-emetic treatment did not occur in the previous cycles.

The number and severity of side-effects were analysed with the Mann–Whitney *U*-test. The number of patients with a rash was compared with Fisher's exact test. All tests were two-tailed using a 5% level of significance.

### RESULTS

In all, 223 consecutive patients were included. A total of 112 patients received granisetron, and 111 received prednisolone plus metopimazine. Patient characteristics are shown in Table 1. Five patients were not eligible because they used benzodiazepines during the daytime, leaving 218 patients evaluable for response in cycle 1.

The results below represent these 218 evaluable patients and do not differ from those obtained in the intention-to-treat analysis of all 223 patients.

In the granisetron group 31, and in the prednisolone plus metopimazine group 32, patients received cyclophosphamide alone during the cycles with concomitant radiotherapy.

Table 2 shows the number of patients 'at risk' and the number withdrawn at each cycle of chemotherapy. Fourteen patients went off study during cycles 1–9 due to concomitant medication with benzodiazepines (one) or steroids (one), termination of chemotherapy before cycle 9 (four), toxicity, especially allergic skin rashes (six) and incorrect study medication (two). The number of cycles evaluable for nausea and vomiting were 514 in the granisetron arm, and 384 in the prednisolone plus metopimazine arm. A reduction in the dose of chemotherapy during cycles 2–9 was performed in 50 (9.7%) cycles in the granisetron group and 37 (9.6%) cycles in the prednisolone plus metopimazine group. Dose reduction was 50% in all but three cycles. All tablets were used in 460 (89.5%) and in 342 (89.1%) of the courses when receiving granisetron, or prednisolone plus metopimazine, respectively. Patients forgot one or more tablets during 32 (6.2%) cycles with granisetron and during 32 (8.3%) cycles with prednisolone plus metopimazine. In 19 (3.7%) and 10 (2.6%) cycles patients were unable to take all tablets because of nausea and vomiting.

### Efficacy of anti-emetic treatment cycle 1

Table 3 shows the response on day 1, days 2–5 and days 1–5. Granisetron was significantly superior to prednisolone plus metopimazine on day 1 ($P < 0.001$) with CR achieved in 75 of the 109 patients (68.8%) compared with 41 of the 109 patients receiving prednisolone plus metopimazine (37.6%).

Among the 218 evaluable patients, six receiving granisetron and 34 prednisolone plus metopimazine were failures on day 1. Of these, one (granisetron) and 16 (prednisolone plus metopimazine)

 © Cancer Research Campaign 1999

**Table 3** Anti-emetic response cycle 1 in 218 patients treated with granisetron or prednisolone plus metopimazine

| | No. of patients (%) | | |
|---|---|---|---|
| | **Granisetron** | **Prednisolone plus metopimazine** | ***P*-value**[a] |
| Day 1 | 109 (100.0) | 109 (100.0) | <0.001 |
| Complete | 75 (68.8) | 41 (37.6) | |
| Major | 18 (16.5) | 21 (19.3) | |
| Minor | 10 (9.2) | 13 (11.9) | |
| Failure | 6 (5.5) | 34 (31.2) | |
| Days 2–5[b] | 107 (100.0) | 93 (100.0) | 0.002 |
| Complete | 54 (50.5) | 65 (69.9) | |
| Major | 26 (24.3) | 18 (19.4) | |
| Minor | 12 (11.2) | 9 (9.7) | |
| Failure | 15 (14.0) | 1 (1.1) | |
| Days 1–5[b] | 109 (100.0) | 109 (100.0) | 0.009 |
| Complete | 49 (45.0) | 35 (32.1) | |
| Major | 26 (23.9) | 24 (22.0) | |
| Minor | 17 (15.6) | 16 (14.7) | |
| Failure | 17 (15.6) | 34 (31.2) | |

[a]Mann–Whitney *U*-test. [b]Based on the worst day in the period. Results based on the total number of emetic episodes and the worst day of nausea in the period are similar.

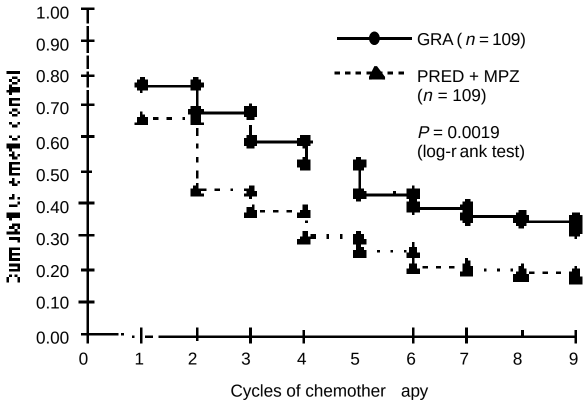

**Figure 1** Maintenance of emetic control in patients with breast cancer treated with granisetron or prednisolone plus metopimazine during nine cycles of adjuvant chemotherapy. GRA = granisetron, PRED = prednisolone, MPZ = metopimazine went off study after day 1 and one granisetron patient was withdrawn after day 1 as she was not satisfied with the anti-emetic treatment. This means that 200 patients were evaluable on days 2–5; 107 in the granisetron group (of which five were failures on day 1) and 93 in the prednisolone plus metopimazine group (of which 18 were failures on day 1). In contrast to day 1, metopimazine plus prednisolone was more effective than granisetron on days 2–5 after chemotherapy. Of the patients with CR on day 1, 65.3% treated with granisetron and 85.4% treated with prednisolone plus metopimazine, also had a CR on days 2–5 ($P$ = 0.029). Among the patients without CR on day 1, 15.6% given granisetron and 57.7% given the combination had CR on days 2–5 ($P < 0.001$). Based on the worst day in the period, CR was observed in 54 of the 107 patients receiving granisetron (50.5%) and in 65 of the 95 patients receiving the combination treatment (69.9%) ($P$ = 0.002, Table 3). Focusing on the overall response, based on the worst day during days 1–5, granisetron was superior to prednisolone plus metopimazine ($P$ = 0.009).

### Efficacy of anti-emetic treatments during multiple cycles

The median number of cycles completed with granisetron was five (95% confidence interval 4–6) compared with two (95% confidence interval 2–2) for prednisolone plus metopimazine ($P$ = 0.0019). The anti-emetic efficacy (cumulative emetic control) of both treatments decreased from cycle 1 to cycle 9 as shown in Figure 1. The number of evaluable patients starting each new course of chemotherapy and the number of patients who were failures, who were not satisfied with the anti-emetic treatment, or who went off study due to other reasons during the nine chemotherapy courses, are given in Table 2. Of the 109 patients in each group, only 28 (25.7%) in the granisetron group compared with 18 (16.5%) in the prednisolone plus metopimazine group completed all nine courses with less than five emetic episodes on any day and were still satisfied with the treatment. Forty patients (36.7%) were failures in the granisetron group compared with 79 (72.3%) in the other group. The number of patients who went off study because they were not satisfied with the anti-emetic treatment were 30 (27.5%, granisetron) and nine (8.3%, prednisolone plus metopimazine).

### Anticipatory nausea and vomiting during cycles 1–9

With granisetron and prednisolone plus metopimazine the number of cycles evaluable for anticipatory nausea and vomiting was 466 and 336 respectively. CR on days 1–3 before the next cycle of chemotherapy was observed in 456 cycles (97.9%) with granisetron and 328 cycles (97.6%) with prednisolone plus metopimazine. Data for anticipatory nausea and vomiting were incomplete in three cycles in both regimens.

© Cancer Research Campaign 1999 

### Safety

In all, 533 cycles with granisetron and 415 cycles with prednisolone plus metopimazine were evaluable for adverse events. These were generally mild to moderate in severity. Based on the worst day during the total number of courses the most frequently reported adverse events in the granisetron group and in the metopimazine plus prednisolone group (% of patients) were headache (40.2% vs 30.6%, $P$ = 0.208), dizziness (16.1% vs 16.3%, $P$ = 0.935), constipation (24.1% vs 2.7%, $P < 0.001$), palpitation (1.8% vs 10.8%, $P$ = 0.015) and rash (11.6% vs 3.6%, $P$ = 0.043). Six patients went off study due to side-effects: four treated with granisetron and two treated with prednisolone plus metopimazine. Among the four patients treated with granisetron, one had an allergic reaction during the infusion of methotrexate in cycles 6 and 7, one had a rash and severe dyspnoea in cycle 3 on day 1, one had a rash and mild dyspnoea in cycle 2 on day 2, and one had a rash in cycle 3 on day 2. One patient treated with prednisolone plus metopimazine went off study due to a rash in cycles 2 and 3, and one due to influenza-like symptoms and severe headache in cycle 1 on day 1 and epigastric pain on days 2–5.

### DISCUSSION

This is the first randomized, double-blind study to investigate anti-emetic efficacy during nine cycles of moderately emetogenic chemotherapy. Granisetron was superior to the combination of prednisolone plus metopimazine in the treatment of acute nausea and vomiting during the first cycle of chemotherapy. CR on day 1 was obtained in 68.8% of the patients treated with granisetron in accordance with the results in other trials investigating a 5-hydroxytryptamine$_3$ (5-HT$_3$) antagonist (Bonneterre et al, 1990; Kaasa et al, 1990; Marty, 1990; Jones et al, 1991; Marschner et al, 1991; Warr et al, 1991; Italian Group for Antimetic Research, 1995*b*).

When the trial was initiated granisetron was available in Denmark for intravenous use only. It has now been shown that a single oral dose of 2 mg granisetron is as effective as intravenous treatment with a serotonin antagonist (Perez et al, 1998).

The efficacy of a corticosteroid plus a dopamine D$_2$ antagonist in the prophylaxis of emesis from moderately emetogenic chemotherapy has been verified in several trials. In comparison with a 5-HT$_3$ antagonist, such a combination equalled (Levitt et al, 1993) or was inferior (Marty, 1990; Warr et al, 1991) to the 5-HT$_3$ antagonist. On the other hand, when the dose of the corticosteroid was increased and the dose divided, the steroid was as effective as a 5-HT$_3$ antagonist (Marschner et al, 1991; Italian Group for Antiemetic Research, 1995*b*). Only a few investigators have addressed the importance of dose and schedule of steroids (Chiara et al, 1987; Coleman et al, 1991; Gez et al, 1992; Havsteen and Kjær, 1996) and the optimum dose and schedule is still unknown. The comparative data, however, suggest that the efficacy of steroids is optimized by divided doses.

Delayed emesis, initially observed after cisplatin, also occurs after moderately emetogenic chemotherapy. Patients receiving non-cisplatin chemotherapy are, however, not always treated for delayed emesis (Marty, 1990; Italian Group for Antiemetic Research, 1995*b*; Silva et al, 1996), as relatively good anti-emetic control is possible during the days following chemotherapy without anti-emetic treatment. Therefore, and because in Denmark granisetron was available for intravenous treatment only when the study was initiated, granisetron was given as a single intravenous dose.

Incomplete protection from acute nausea and vomiting is the most important risk factor for delayed emesis after cisplatin (Roila et al, 1991; Italian Group for Antiemetic Research, 1994). However, in one trial, in-patient status was the only significant risk factor for delayed emesis after moderately emetogenic chemotherapy (Kaizer et al, 1994). We observed that a 3-day treatment with prednisolone plus metopimazine was superior to a single dose of granisetron in the treatment of delayed emesis in cycle 1. Only one patient receiving the combination failed on days 2–5, even though 18 of the 93 patients evaluated for delayed emesis were failures on day 1. In the granisetron group, six patients were failures on day 1, and on days 2–5, 15 patients were failures. Furthermore, among both patients with CR and patients with non-complete response day 1 in the initial cycle the protection from delayed nausea and vomiting was significantly superior with prednisolone plus metopimazine.

Our results indicate that the combination of a corticosteroid plus a dopamine D$_2$ antagonist is effective in the treatment of delayed emesis following moderately emetogenic chemotherapy. This is in accordance with other studies where low-dose metoclopramide plus dexamethasone was more effective than either placebo (Kris et al, 1989) or dexamethasone alone (Kris et al, 1989; Moreno et al, 1992), dexamethasone plus prochlorpromazine more effective than granisetron (Matsui et al, 1996), and alizapride more effective than the 5-HT$_3$ antagonist ondansetron (Münstedt et al, 1995) in controlling delayed nausea and vomiting after cisplatin. After cyclophosphamide-based chemotherapy dexamethasone alone is effective (Koo and Ang, 1996). In trials evaluating the efficacy of anti-emetic agents during multiple cycles of moderately emetogenic chemotherapy (non-cisplatin – Blijham, 1992; Martin et al, 1992; Soukop et al, 1992; Italian Group for Antiemetic Research, 1995*a*; Silva et al, 1996; non-cisplatin and cisplatin < 50 mg $m^{-2}$ – de Wet et al, 1993; Kaizer et al, 1994) several diversities in patient population (Blijham, 1992; Martin et al, 1992; Soukop et al, 1992; de Wet et al, 1993; Kaizer et al, 1994; Italian Group for Antiemetic Research, 1995*a*; Silva et al, 1996), trial methodology (Blijham, 1992; Martin et al, 1992; Soukop et al, 1992; de Wet et al, 1993; Kaizer et al, 1994; Italian Group for Antiemetic Research, 1995*a*; Silva et al, 1996), or statistical analyses complicate the interpretation of results. Some authors include all patients in the evaluation of anti-emetic effect during multiple cycles (Martin et al, 1992; Soukop et al, 1992; Italian Group for Antiemetic Research, 1995*a*; Silva et al, 1996), whereas others include only patients with 'good' anti-emetic response during the first cycle of chemotherapy (de Wet et al, 1993; Kaizer et al, 1994) or only those who requested the same anti-emetic treatment in the following cycles (Blijham, 1992). It has been stated that the Kaplan–Meier method should be used for the calculation of the overall protection during multiple cycles (De Wit et al, 1996). If calculations are based on conditional probabilities of protection, only patients with protection in previous cycles are included leading to an overestimation of the sustainment of protection (De Wit et al, 1996). Using the Kaplan–Meier method we found that the efficacy of both treatments decreased during multiple cycles, but with the method of conditional probabilities the efficacy increased (Table 2). Some trials have reported sustained efficacy of anti-emetic treatment during multiple cycles of moderately emetogenic chemotherapy (Blijham, 1992; de Wet et al, 1993; Kaizer et al, 1994; Italian Group for Antiemetic Research, 1995*a*; Silva et al, 1996), but in three of these trials the investigators used the method of conditional probabilities for the calculation (Blijham, 1992; de Wet et al,

 © Cancer Research Campaign 1999

1993; Kaizer et al, 1994). In two of the studies the number of patients still at risk decreased to less than 10% in cycles 8 and 10 respectively (Blijham, 1992; de Wet et al, 1993) and in the other three (Kaizer et al, 1994; Italian Group for Antiemetic Research, 1995*a*; Silva et al, 1996) the efficacy was only evaluated during the first three cycles. The trials reporting decreasing efficacy of anti-emetic treatment during multiple cycles evaluated the efficacy during six cycles of chemotherapy (Martin et al, 1992; Soukop et al, 1992). As in our study, only women were included. Females are at higher risk of developing nausea and vomiting after chemotherapy than men (Tonato et al, 1991). The differences in study design and statistical methods may explain the different results when evaluating anti-emetic efficacy during multiple cycles. To detect a decrease in efficacy of anti-emetic efficacy, three cycles may be too few. The reason for this decrease of efficacy during multiple cycles is unknown. Andrews et al have demonstrated, in ferrets treated with radiotherapy, 'plasticity and modulation' of the emetic pathway following vagotomy (Andrews and Davis, 1993) and the decrease in anti-emetic efficacy during multiple cycles might be explained by this 'plasticity' or reorganization in the emetic pathway.

Both treatments were generally well-tolerated. As in other studies, constipation was more frequently reported with granisetron. In previous trials, rash has rarely been reported with granisetron (Adams and Valley, 1995). However, in this study 13% of patients treated with granisetron reported rash. It is possible that this was caused by granisetron or by concomitant medication including chemotherapy. Many trials have only followed the patients on day 1 during the initial cycle. Except for two patients in our trial, rash was not reported during cycle 1, but during cycle 2 or the following cycles. With observation of patients for more than one day and after cycle 1, rash may be reported more frequently.

The efficacy of granisetron and of prednisolone plus metopimazine declined during nine cycles of CMF/CEF chemotherapy and after completion of cycle 9 only 25.7% and 16.5% of patients, respectively, were still at risk. This clearly emphasizes the need for improvement of anti-emetic efficacy. A combination of both a 5-$HT_3$ antagonist, a steroid and a dopamine $D_2$ antagonist might further improve the control of nausea and vomiting induced by moderately emetogenic chemotherapy. This is supported by investigations showing that the efficacy of a 5-$HT_3$ antagonist is improved by addition of a steroid (Italian Group for Antiemetic Research, 1995*b*) and metopimazine (Herrstedt et al, 1993). Future trials investigating the efficacy of a 5-$HT_3$ antagonist in combination with either a steroid or a dopamine $D_2$ antagonist or both during multiple cycles of chemotherapy are warranted.

### ACKNOWLEDGEMENTS

Supported by Smithkline Beecham A/S and Rhone-Poulenc Rorer A/S.

### REFERENCES

Aapro MS (1991) Controling emesis related to cancer therapy. *Eur J Cancer* **27**: 356–361

Adams VR and Valley AW (1995) Granisetron: the second serotonin-receptor antagonist. *Ann Pharmacother* **29**: 1240–1251

Andrews PLR and Davis CJ (1993) The mechanism of emesis induced by anticancer therapies. In *Emesis in Anticancer Therapy*, 1st Edn, Andrews PLA and Sanger GJ (eds), pp. 113–161. Chapman & Hall: London

Blijham HG (1992) Does granisetron remain effective over multiple cycles? *Eur J Cancer* **28A**: S17–S21

Bonneterre J, Chevallier B, Metz R, Fargeot P, Pujade-Laurine E, Spielmann M, Tubiana-Hulin M, Paes D and Bons J (1990) A randomized double-blind comparison of ondansetron and metoclopramide in the prophylaxis of emesis induced by cyclophosphamide, fluorouracil, and doxorubicin or epirubicin chemotherapy. *J Clin Oncol* **8**: 1063–1069

Campora E, Simoni C and Rosso R (1994) Tropisetron verso ondansetron nella preventione e controllo dellémesi in pazienti sottoposte a chemioterapia con FAC/FEC per carcinoma mammario metastatico o operato. *Minerva Med* **85**: 25–31

Cersosimo RJ and Karp DD (1986) Adrenal corticosteroids as antiemetics during cancer chemotherapy. *Pharmacotherapy* **6**: 118–127

Chiara S, Campora E, Lionetto R, Bruzzi P and Rosso R (1987) Methylprednisolone for the control of CMF-induced emesis. *Am J Clin Oncol* **10**: 264–267

Clavel M, Soukop M and Greenstreet YLA (1993) Improved control of emesis and quality of life with ondansetron in breast cancer. *Oncology* **50**: 180–183

Coleman RE, Twelves CJ, O'Reilly SM, Rubens RD, Richards MA and Harper PG (1991) Influence of dexamethasone dose on the control of chemotherapy-induced nausea and vomiting. *Eur J Cancer* **27**: 1062–1063

de Wet M, Falkson G and Rapoport L (1993) Repeated use of granisetron in patients receiving cytostatic agents. *Cancer* **71**: 4043–4049

De Wit R, Schmitz PIM, Verweij J, De Boer-Dennart M, De Mulder PHM, Planting AST, Van Der Burg MEL and Stoter G (1996) Analysis of cumultative probabilities shows that the efficacy of 5-HT3 antagonist prophylaxis is not maintained. *J Clin Oncol* **14**: 644–651

Early Breast Cancer Trialists' Collaborative Group (1992) Systemic treatment of early breast cancer by hormonal, cytotoxic, or immune therapy. *Lancet* **339**: 71–85

Gebbia V, Cannata G, Testa A, Curto G, Valenza R, Cipolla C, Latteri MA and Gebbia N (1994) Ondansetron versus granisetron in the prevention of chemotherapy-induced nausea and vomiting. *Cancer* **74**: 1945–1952

Gez E, Strauss N, Vitzhaki N, Cass Y and Edelmann DZ (1992) Methylprednisolone as antiemetic treatment in breast-cancer patients receiving cyclophosphamide, methotrexate, and 5-fluorouracil: a prospective, crossover, randomized blind study comparing two different dose schedules. *Cancer Chemother Pharmacol* **30**: 229–232

Havsteen H and Kjær M (1996) Antiemetic treatment with two different doses of methylprednisolone in breast cancer patients: a double-blind randomized crossover study with evaluation of efficacy parameters. *Anti-Cancer Drugs* **7**: 535–542

Herrstedt J, Sigsgaard T, Boesgaard M, Jensen TP and Dombernowsky P (1993) Ondansetron plus metopimazine compared with ondansetron alone in patients receiving moderately emetogenic chemotherapy. *N Engl J Med* **328**: 1076–1080

Herrstedt J, Sigsgaard T, Angelo HR, Kampmann JP and Hansen M (1997) Dose-finding study of oral metopimazine. *Support Care Cancer* **5**: 38–43

Ibrahim EM, Al-Idrissi HY, Ibrahim A, Absood G, Al-Dossary E, Al-Jammaa A, Al-Ethan S and Eliopoulos A (1986) Antiemetic efficacy of high-dose dexamethasone: Randomized, double-blind, crossover study with high-dose metoclopramide in patients receiving cancer chemotherapy. *Eur J Cancer Clin Oncol* **22**: 283–288

Israel L and Rodary C (1978) Treatment of nausea and vomiting related to anti-cancerous multiple combination chemotherapy: results of two controlled studies. *J Int Med Res* **6**: 235–240

Italian Group for Antiemetic Research (1994) Cisplatin-induced delayed emesis: pattern and prognostic factors during three subsequent cycles. *Ann Oncol* **5**: 585–589

Italian Group for Antiemetic Research (1995*a*) Persistence of efficacy of three antiemetic regimens and prognostic factors in patients undergoing moderately emetogenic chemotherapy. *J Clin Oncol* **13**: 2417–2426

Italian Group for Antiemetic Research (1995*b*) Dexamethasone, granisetron, or both for the prevention of nausea and vomiting during chemotherapy for cancer. *N Engl J Med* **332**: 1–5

Jones A, Hill AS, Soukop M, Hutcheon AW, Cassidy J, Kaye SB, Sikora K, Carney DN and Cunningham D (1991) Comparison of dexamethasone and ondansetron in the prophylaxis of emesis induced by moderately emetogenic chemotherapy. *Lancet* **338**: 483–487

Kaasa S, Kvaløy S, Dicato MA, Ries F, Huys JV, Royer E and Carruthers L (1990) A comparison of ondansetron with metoclopramide in the prophylaxis of chemotherapy-induced nausea and vomiting: A randomized, double-blind study. *Eur J Cancer* **26**: 311–314

Kaizer L, Warr D, Hoskins P, Latreille J, Lofters W, Yau J, Palmer M, Zee B, Levy M and Pater J (1994) Effect of schedule and maintenance on the antiemetic

© Cancer Research Campaign 1999

efficacy of ondansetron combined with dexamethasone in acute and delayed nausea and emesis in patients receiving moderately emetogenic chemotherapy: a phase III trial by the National Cancer Institute of Canada Clinical Trials Group. *J Clin Oncol* **12**: 1050–1057

Koo WH and Ang PT (1996) Role of maintenance oral dexamethasone in prophylaxis of delayed emesis caused by moderately emetogenic chemotherapy. *Ann Oncol* **7**: 71–74

Kris MG, Gralla RJ, Tyson LB, Clark RA, Cirrincione C and Groshen S (1989) Controlling delayed vomiting: double-blind, randomized trial comparing placebo, dexamethasone alone, and metoclopramide plus dexamethasone in patients receiving cisplatin. *J Clin Oncol* **7**: 108–114

Levitt M, Warr D, Yelle L, Rayner HL, Lofters WS, Perrault DJ, Wilson KS, Latreille J, Potvin M, Warner E, Pritchard KI, Palmer M, Zee B and Pater JL (1993) Ondansetron compared with dexamethasone and metoclopramide as antiemetics in the chemotherapy of breast cancer with cyclophosphamide, methotrexate, and fluorouracil. *N Engl J Med* **328**: 1081–1084

Markman M, Sheidler V, Ettinger DS, Quaskey SA and Mellits ED (1984) Antiemetic efficacy of dexamethasone. Randomized, double-blind, crossover study with prochlorperazine in patients receiving cancer chemotherapy. *N Engl J Med* **311**: 549–552

Marschner NW, Adler M, Nagel GA, Christmann D, Fenzl E and Upadhyaya B (1991) Double-blind randomised trial of the antiemetic efficacy and safety of ondansetron and metoclopramide in advanced breast cancer patients treated with epirubucin and cyclophosphamide. *Eur J Cancer* **27**: 1137–1140

Martin M, Diaz-Rubio E, Casado A, Dominguez S and Sastra J (1992) Progressive loss of antiemetic efficacy during subsequent courses of chemotherapy. *Eur J Cancer* **28**: 430–432

Marty M (1990) A comparative study of the use of granisetron, a selective 5-HT3 antagonist, versus a standard enti-emetic regimen of chlorpromazine plus dexamethasone in the treatment of cytostatic-induced emesis. *Eur J Cancer* **26**: S28–S32

Matsui K, Fukuoka M, Takada M, Kusunoki Y, Yana T, Tamura K, Yoshida T, Iida K, Hirashima T, Tsukada H, Ushijima S, Miyawaki H and Masuda N (1996) Randomized trial for the prevention of delayed emesis in patients receiving high-dose cisplatin. *Br J Cancer* **73**: 217–221

Moertel CG and Reitemeier RJ (1973) Controlled studies of metopimazine for the treatment of nausea and vomiting. *J Clin Pharmacol* **13**: 283–287

Moreno I, Rosell R, Abad A, Barnadas A, Carles J, Ribelles N, Solano V and Font A (1992) Comparison of three protracted antiemetic regimens for the control of delayed emesis in cisplatin-treated patients. *Eur J Cancer* **28A**: 1344–1347

Münstedt K, Milch W, Blaunth-Eckmeyer E, Spänle A, Vahrson H and Reimer C (1995) Prevention of cisplatinum-induced delayed emesis and nausea. *Onkologie* **18**: 23–26

Perez EA, Hesketh P, Sandbach J, Reeves J, Chawla S, Markman M, Hainsworth J, Bushnell W and Friedman C (1998) Comparison of single-dose oral granisetron versus intravenous ondansetron in the prevention of nausea and vomiting induced by moderately emetogenic chemotherapy: a multicenter, double-blind, randomized parallel study. *J Clin Oncol* **16**: 754–760

Pollera CF, Nardi M, Marolla P, Pinnaro P, Terzoli E and Giannarelli D (1989) Effective control of CMF-related emesis with high-dose dexamethasone: results of a double-blind crossover trial with metoclopramide and placebo. *Am J Clin Oncol* **12**: 524–529

Roila F, Basurto C, Minotti V, Ballatori E, Tonato M and Del Favero A (1988) Methylprednisolone versus metoclopramide for prevention of nausea and vomiting in breast cancer patients treated with intravenous cyclophosphamide methotrexate 5-fluorouracil: a double-blind randomized study. *Oncology* **45**: 346–349

Roila F, Boschetti E, Tonato M, Basurto C, Bracarda S, Picciafuoco M, Patoia L, Santi E, Penza O, Ballatori E and Del Favero A (1991) Predictive factors of delayed emesis in cisplatin-treated patients and antiemetic activity and tolerability of metoclopramide or dexamethasone. *Am J Clin Oncol* **14**: 238–242

Silva RR, Bascioni R, Giorgi F, Acito L, De Signoribus G, Marcellini M, Menichetti ET and Giuliodori L (1996) Granisetron plus dexamethasone in moderately emetogenic chemotherapy: evaluation of activity during three consecutive courses of chemotherapy. *Support Care Cancer* **4**: 287–290

Soukop M, McQuade B, Hunter E, Stewart A, Kaye S, Cassidy J, Kerr D, Khanna S, Smyth J, Coleman R, Cunningham D, Powels T, Davidson N, Hutcheon A, Green J, Slater A, Rustin G and Carney D (1992) Ondansetron compared with metoclopramide in the control of emesis and quality of life during repeated chemotherapy for breast cancer. *Oncology* **49**: 295–304

Stewart A, McQuade B, Cronje JDE, Goedhals L, Gudgeon A, Corette L, Froger X, Tubiana-Hulin M, Laplaige P, Roberts JT, Mcrae J, Forster J, Parasuraman TV and Butcher M (1995) Ondansetron compared with granisetron in the prophylaxis of cyclophosphamide-induced emesis in out-patients: A multicenter, double-blind, double-dummy, randomised parallel-group study. *Oncology* **52**: 202–210

Tonato M, Roila F and Del Favero A (1991) Methodology of antiemetic trials: A review. *Ann Oncol* **2**: 107–114

Vallejo C, Rabinovich M, Leone B and Gonzalez J (1988) Toxicity and dose-response of intravenous (i.v.) metopimazine (MPZ) as preventive of high-dose cisplatin (CDDP)-induced emesis. *Proc Am Soc Clin Oncol* **7**: 286 (Abstract)

Warr D, Willan A, Fine S, Wilson K, Davis A, Erlichman C, Rusthoven J, Lofters W, Osoba D, Laberge F, Latreille J and Pater J (1991) Superiority of granisetron to dexamethasone plus pro0chlorperazine in the prevention of chemotherapy-induced emesis. *J Natl Cancer Inst* **83**: 1169–1173

World Health Organisation (1979) *Handbook for Reporting Results of Cancer Treatment*. World Health Organisation: Bask

*© Cancer Research Campaign 1999*
